# The *ecl* family gene *ecl3*^+^ is induced by phosphate starvation and contributes to sexual differentiation in fission yeast

**DOI:** 10.1242/jcs.260759

**Published:** 2023-03-10

**Authors:** Hokuto Ohtsuka, Hiroki Sakata, Yuto Kitazaki, Masanobu Tada, Takafumi Shimasaki, Yoko Otsubo, Yasukichi Maekawa, Mikuto Kobayashi, Kazuki Imada, Akira Yamashita, Hirofumi Aiba

**Affiliations:** ^1^ Laboratory of Molecular Microbiology, Graduate School of Pharmaceutical Sciences, Nagoya University, Nagoya, Aichi 464-8601, Japan; ^2^ Interdisciplinary Research Unit, National Institute for Basic Biology, Okazaki, Aichi 444-858, Japan; ^3^ National Institute for Fusion Science, Toki, Gifu 509-5292, Japan; ^4^ Center for Novel Science Initiatives, National Institutes of Natural Sciences, Okazaki, Aichi 444-8585, Japan; ^5^ Department of Chemistry and Biochemistry, National Institute of Technology (KOSEN), Suzuka College, Suzuka 510-0294, Japan; ^6^ Department of Biology, Graduate School of Science, Osaka City University, Sumiyoshi-ku, Osaka 558-8585, Japan; ^7^ Center for Low-temperature Plasma Sciences, Nagoya University, Nagoya, Aichi 464-8603, Japan

**Keywords:** *ecl* family genes, G1 cell cycle arrest, Phosphate starvation, Sexual differentiation, Phosphate signal transduction pathway, Fission yeast, Lifespan

## Abstract

In *Schizosaccharomyces pombe*, *ecl* family genes are induced by several signals, such as starvation of various nutrients, including sulfur, amino acids and Mg^2+^, and environmental stress, including heat or oxidative stress. These genes mediate appropriate cellular responses and contribute to the maintenance of cell viability and induction of sexual differentiation. Although this yeast has three *ecl* family genes with overlapping functions, any environmental conditions that induce *ecl3*^+^ remain unidentified. We demonstrate that *ecl3*^+^ is induced by phosphate starvation, similar to its chromosomally neighboring genes, *pho1*^+^ and *pho84*^+^, which respectively encode an extracellular acid phosphatase and an inorganic phosphate transporter. *ecl3*^+^ expression was induced by the transcription factor Pho7 and affected by the cyclin-dependent kinase (CDK)-activating kinase Csk1. Phosphate starvation induced G1 arrest and sexual differentiation via *ecl* family genes. Biochemical analyses suggested that this G1 arrest was mediated by the stabilization of the CDK inhibitor Rum1, which was dependent on *ecl* family genes. This study shows that *ecl* family genes are required for appropriate responses to phosphate starvation and provides novel insights into the diversity and similarity of starvation responses.

## Teaser

**Summary:** The genes of the fission yeast *ecl* family respond to various forms of starvation. Phosphate starvation induces *ecl3*^+^, which contributes to starvation-induced G1 arrest and sexual differentiation.

## INTRODUCTION

There are three *ecl* family genes in *Schizosaccharomyces pombe*, namely, *ecl1*^+^, *ecl2*^+^ and *ecl3*^+^, which encode small proteins composed of less than 100 amino acids that are predicted to bind Zn^2+^ through conserved cysteine residues (Ohtsuka and Aiba, 2017; Shimasaki et al., 2017). These genes act on the extension of chronological lifespan (CLS), which is defined as the survival period of the nondividing cell population, measured by the viability after entry into the stationary phase in yeast (Naito et al., 2014; Masumura et al., 2019; Lee et al., 2021; Romila et al., 2021; Ohtsuka et al., 2021b, 2022c; Legon and Rallis, 2022). These genes are conserved in fungi, including *Saccharomyces cerevisiae* (Azuma et al., 2012; Ohtsuka and Aiba, 2017; Zhang et al., 2021). In *S. pombe*, *ecl* family genes are induced by various signals, including starvation of the nitrogen source, sulfur source, amino acids and Mg^2+^, and then induce CLS extension, sexual differentiation and autophagy (Miwa et al., 2011; Shimasaki et al., 2020; Ohtsuka et al., 2021a,c, 2022b). The Ecl family proteins also inhibit the activity of target of rapamycin complex 1 (TORC1) and contribute to the regulation of the cell cycle under starvation (Ohtsuka et al., 2022a). Under sulfur starvation, *ecl1*^+^ is induced by the transcription factor Zip1 (Ohtsuka et al., 2017). Under starvation of amino acids or Mg^2+^, the transcription factor Fil1 induces *ecl1*^+^ through the general amino acid control system (Ohtsuka et al., 2019, 2021a). Oxidative stress induces *ecl1*^+^ through the transcription factor Atf1 (Shimasaki et al., 2014). Heat stress induces *ecl2*^+^ through the heat shock transcription factor Hsf1 (Ohtsuka et al., 2011). However, although several conditions that induce *ecl1*^+^ and *ecl2*^+^ have been identified, any environmental conditions inducing *ecl3*^+^ have not been clarified.

In *S. pombe*, intracellular phosphate levels are maintained by the phosphate signal transduction pathway (PHO pathway), which regulates gene expression in response to phosphate starvation (Henry et al., 2011; Estill et al., 2015; Zhou et al., 2016). The PHO pathway is regulated by three phosphate uptake genes that are primarily controlled by the phosphate homeostasis regulon (Garg et al., 2022): *pho1*^+^, which encodes a cell-surface acid phosphatase, a secreted glycoprotein required for extracellular phosphate uptake (Schweingruber et al., 1992); *pho84*^+^, which encodes an inorganic phosphate transporter, one of five phosphate transporters in *S. pombe* (Sawada et al., 2021); and *tgp1*^+^, which encodes a glycerophosphate transporter (Garg et al., 2018a). It has been reported that the degradation of phosphate transporters is also involved in the maintenance of intracellular phosphate homeostasis, e.g. SPX-RING-type ubiquitin ligase Pqr1 regulates phosphate uptake through the ubiquitination and degradation of phosphate transporters, including Pho84 (Sawada et al., 2021). According to studies, phosphate starvation induces the phosphorylation of the stress-response mitogen-activated kinase Sty1 and causes autophagy (Zuin et al., 2010; Corral-Ramos et al., 2022).

The Zn_2_Cys_6_ transcription factor Pho7 is a crucial factor acting on the response to phosphate starvation (Estill et al., 2015; Schwer et al., 2017; Garg et al., 2019). Pho7 binds to the 5′-TCG(G/C)(A/T)xxTTxAA-3′ DNA motif, but no ortholog has been identified in *S. cerevisiae* (Vishwanatha et al., 2016; Schwer et al., 2017). Pho7 regulates the expression of genes involved in the PHO pathway, including *pho1*^+^, *pho84*^+^ and *tgp1*^+^ (Carter-O'Connell et al., 2012; Schwer et al., 2017). The expression of *pho1*^+^, *pho84*^+^ and *tgp1*^+^ is regulated by the PHO regulon, and their transcription is negatively regulated by 5′-flanking interfering upstream long noncoding RNAs (lncRNAs), namely, *nc-pho1*/*prt* (*pho*-repressive transcript), *prt2* and *nc-tgp1*, respectively (Shah et al., 2014; Sanchez et al., 2018; Garg et al., 2020; Shuman, 2020). The PHO regulon is activated by slowly transcribing RNA polymerase II (Pol II) through a mechanism involving alternative polyadenylation and premature transcription termination of lncRNAs (Yague-Sanz et al., 2020). Additionally, it has been suggested that the expression of lncRNAs that regulate PHO genes is regulated by the HomolD box (Garg et al., 2018b). The HomolD-binding protein Rrn7 forms a complex with Pol II, and the phosphorylation of Thr67 of Rrn7 is regulated by casein kinase 2; the phosphorylation of Rrn7 by casein kinase 2 suppresses transcriptional activity regulated by HomolD (Moreira-Ramos et al., 2015; Montes et al., 2017).

In this study, we sought to further understand the relationship between the *ecl* family genes and starvation response; in addition, we aimed to demonstrate that *ecl3*^+^ is induced by phosphate starvation with a mechanism similar to that of the PHO regulon and contributes to the sexual differentiation in *S. pombe*. The *ecl* family genes regulate appropriate G1 cell cycle arrest by stabilizing the cyclin-dependent kinase (CDK) inhibitor Rum1 and then induce sexual differentiation under phosphate starvation. Three genes, *pho1*^+^, *pho84*^+^ and *tgp1*^+^, are regulated by the PHO regulon and involved in the cellular response to phosphate starvation. Our data suggest that, similar to these genes, *ecl3*^+^ is also integrated as a member of the PHO regulon and contributes to appropriate cellular responses to phosphate starvation in *S. pombe*.

## RESULTS

### Phosphate starvation induces *ecl3*^+^ in a Pho7-dependent manner

**Fig. 1.**
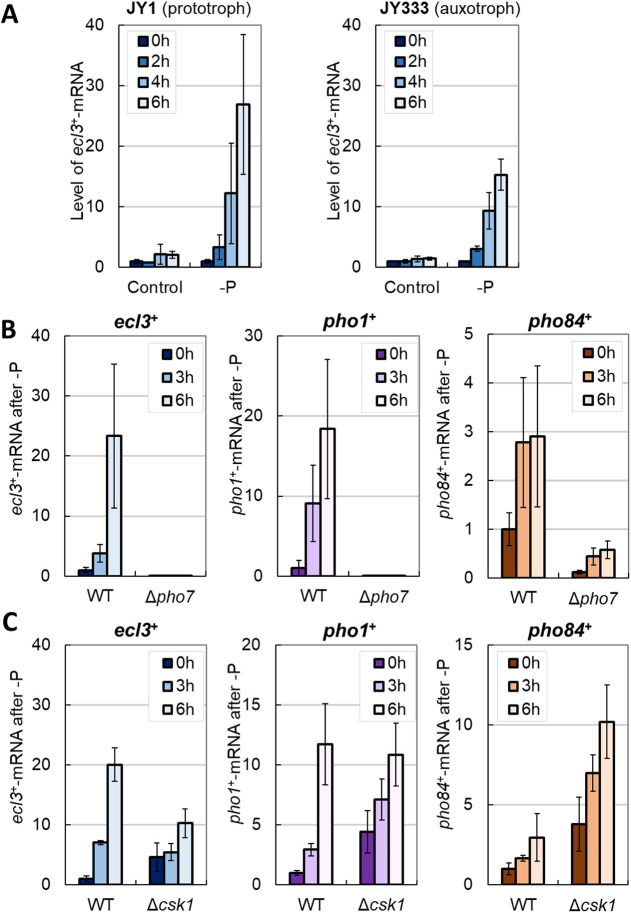
**Phosphate starvation induces *ecl3*^+^ expression in a *pho7*^+^-dependent manner.** The mRNA levels of *ecl3*^+^, *pho1*^+^ and *pho84*^+^ were measured by real-time PCR. (A) Prototrophic JY1 and auxotrophic JY333 cells were grown in Edinburgh minimal medium (EMM) to OD_600_=0.5 and then transferred into EMM with (control) and without Na_2_HPO_4_ (−P) (*n*=3). The expression of *ecl3*^+^ at 0, 2, 4 and 6 h after phosphate starvation was measured. (B) JY333 (wild-type or WT) and JY333Δ*pho7* (Δ*pho7*) cells were grown in EMM to OD_600_=0.5 and then transferred into EMM without Na_2_HPO_4_ (*n*=3). The expression of *ecl3*^+^, *pho1*^+^ and *pho84*^+^ at 0, 3 and 6 h after phosphate starvation was measured. (C) JY333 (WT) and JY333Δ*csk1* (Δ*csk1*) cells were grown in EMM to OD_600_=0.5 and then transferred into EMM without Na_2_HPO_4_ (*n*=3). The expression of *ecl3*^+^, *pho1*^+^ and *pho84*^+^ at 0, 3 and 6 h after phosphate starvation was measured. For cultures of JY333, JY333Δ*pho7* and JY333Δ*csk1*, 40 mg l^−1^ adenine and 60 mg l^−1^ leucine were added. Data show the mean±s.d.

It has been reported that *ecl3*^+^ is upregulated in the *asp1* mutant *asp1-H397A*, which encodes a kinase synthesizing inositol pyrophosphate (IPP) (Sanchez et al., 2019). IPP is a signal molecule regulating phosphate homeostasis in *S. cerevisiae* and *Arabidopsis thaliana*, and probably also in *S. pombe* (Sanchez et al., 2019; Shuman, 2020; Benjamin et al., 2022). *pho84*^+^ and *pho1*^+^ are present on chromosome II in order, immediately adjacent to the 5′ end of *ecl3*^+^. It has been proposed that the upregulation of *ecl3*^+^ in the *asp1* mutant reflects its proximity to *prt2*, which regulates *pho84*^+^; the *prt2* promoter might be bidirectional, and lncRNAs that are transcribed in the opposite direction to *prt2* might interfere with *ecl3*^+^ transcription (Sanchez et al., 2019). However, it is unclear whether the induction of *ecl3*^+^ is physiologically significant or merely a secondary effect for *pho84*^+^ regulation, and whether phosphate starvation itself also induces *ecl3*^+^. We first examined the expression level of *ecl3^+^* to confirm whether phosphate depletion itself regulates *ecl3*^+^ expression. Results showed that phosphate starvation itself also increased *ecl3*^+^ expression (Fig. 1A). Moreover, we observed the induction of *ecl3*^+^ irrespective of the presence or absence of auxotrophy.

We next investigated the mechanism that regulates *ecl3*^+^ induction. According to studies, genes adjacent to *ecl3*^+^ on chromosome II, namely *pho1*^+^ and *pho84*^+^, are regulated by the transcription factor Pho7 and the CDK-activating protein Csk1 (Carter-O'Connell et al., 2012; Schwer et al., 2017). Furthermore, a previous chromatin immunoprecipitation experiment coupled with high-throughput sequencing analysis demonstrated that Pho7 binds upstream of not only *pho1*^+^ and *pho84*^+^ but also *ecl3*^+^ (Carter-O'Connell et al., 2012). Therefore, we explored whether *ecl3*^+^ expression is regulated by Pho7 and Csk1. Phosphate starvation induced the expression of *ecl3*^+^, *pho1*^+^ and *pho84*^+^ in a Pho7-dependent manner (Fig. 1B), suggesting that Pho7 directly regulates *ecl3*^+^ expression under phosphate starvation. Although studies have also reported that Csk1 functions upstream of Pho7 and negatively regulates the expression of *pho1*^+^ and *pho84*^+^ (Henry et al., 2011; Carter-O'Connell et al., 2012; Estill et al., 2015), the deletion of *csk1*^+^ increased the expression of *ecl3*^+^ as well as that of *pho1*^+^ and *pho84*^+^ (Fig. 1C). These data suggest that *ecl3*^+^, similar to its neighboring genes, *pho1*^+^ and *pho84*^+^, was also subjected to transcriptional control by Pho7 and Csk1.

Previous studies have reported that the mRNA level of *ecl3*^+^ is also negatively regulated by the zinc finger protein Zfs1, which regulates mRNA catabolism and sexual differentiation (Goldar et al., 2005; Wells et al., 2012; Navarro et al., 2017). We next investigated the relationship between Zfs1 and *ecl3*^+^ induction by phosphate starvation. Although Zfs1 is expected to bind *ecl3*^+^ transcripts and regulate their levels, the deletion of *zfs1*^+^ increased the mRNA level of *ecl3*^+^ only in yeast extract (YE) complete medium and not in Edinburgh minimal medium (EMM) (Fig. S1A,B). In addition, phosphate starvation increased the mRNA level of *ecl3*^+^ with or without Zfs1 (Fig. S1B). These results suggest that Zfs1 is involved in the repression of the *ecl3*^+^ transcript levels in a nutrient-rich environment but is not required for induction by phosphate starvation.

Next, we examined how phosphate starvation would affect the expression of other *ecl* family genes. We measured the expression of *ecl1*^+^ and *ecl2*^+^ during phosphate starvation using *pho7*^+^- or *csk1*^+^-deficient strains (Fig. S2A,B). In *S. pombe* wild-type strains, the expression of *ecl1*^+^ and *ecl2*^+^ was not dramatically induced by phosphate starvation, as seen for *ecl3*^+^. In contrast, the deletion of *pho7*^+^ or *csk1*^+^ significantly increased the expression of *ecl1*^+^. Studies have reported that *ecl1*^+^ is expressed in response to various stimuli by multiple transcription factors, including the transcription factors Atf1, Zip1 and Fil1 (Shimasaki et al., 2014; Ohtsuka et al., 2017, 2019). It has also been reported that *ecl1*^+^ is subject to gene silencing by the Erh1–Mmi1 complex (Xie et al., 2019), which regulates meiotic mRNA decay and heterochromatin assembly (Sugiyama et al., 2016; Wei et al., 2021). Consistent with this report, a temperature-sensitive mutant of *mmi1*^+^ exhibited high expression of *ecl1*^+^ under restrictive temperature (Fig. S2C). It is currently unknown whether the upregulation of *ecl1*^+^ by *pho7*^+^ or *csk1*^+^ deficiency is direct or indirect through other transcription factors or gene silencing. Taken together, these data indicate that phosphate starvation primarily induces the expression of *ecl3*^+^ among the *ecl* family genes in a Pho7-dependent manner.

### *ecl* family genes do not suppress the stress sensitivities and resistances of Δ*pho7* cells

Previous studies have reported that the Δ*pho7* strain exhibits temperature sensitivity, cold sensitivity, and resistance to 2-deoxyglucose (2-DG) and H_2_O_2_ (Vishwanatha et al., 2016, 2017; Schwer et al., 2017). We investigated the growth of the Δ*pho7* and Δ*ecl1/2/3* (Δ*ecls*) strains under these stress conditions. Results showed that Δ*pho7* cells exhibited weak growth retardation under high temperature and strong 2-DG resistance, whereas Δ*ecls* cells showed no significant differences from wild-type cells (Fig. S3A,B). However, both Δ*pho7* and Δ*ecls* cells exhibited growth retardation under low temperature conditions. Nevertheless, these sensitivities of Δ*pho7* cells to high or low temperature were not suppressed by *ecl3*^+^ expression in cells carrying the pEcl3 plasmid, which expressed *ecl3*^+^ at a level equal to or higher than that in wild-type cells, even in Δ*pho7* cells (Fig. S3A,C). This result suggests that these temperature sensitivities of Δ*pho7* cells are not due to reduced *ecl3*^+^ expression. Δ*pho7* cells were resistant to H_2_O_2_ stress, whereas Δ*ecls* cells were sensitive, consistent with a previous report (Shimasaki et al., 2014). Overexpression of *ecl3*^+^ resulted in sensitivity to 2-DG, but *ecl3*^+^ expression in Δ*pho7* cells did not suppress the resistance to 2-DG (Fig. S3B). Based on these results, we could not identify any strong connection between the *ecl* family gene and *pho7*^+^ in these stress responses.

### Ckb1 is required for proper *ecl3*^+^ induction under phosphate starvation

**Fig. 2.**
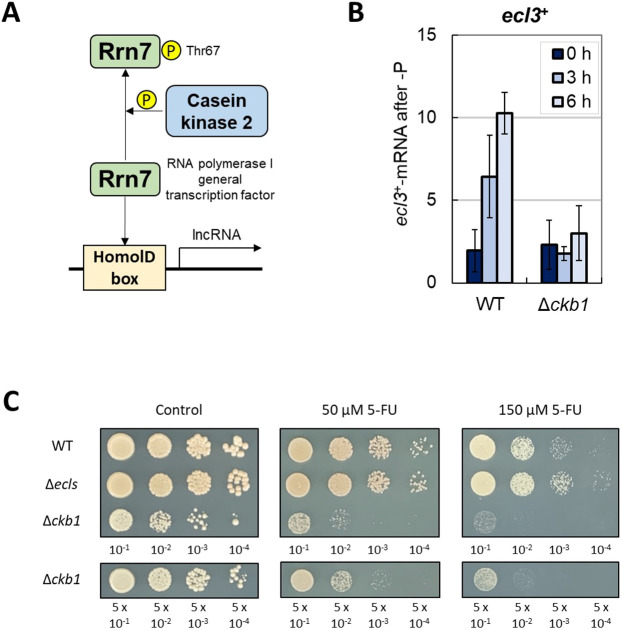
**Casein kinase 2 regulates proper *ecl3*^+^ induction under phosphate starvation.** (A) Simple illustration of the regulation of lncRNA expression via the HomolD box. Phosphorylation of Rrn7 by casein kinase 2 prevents the binding of Rrn7 to the HomolD box. (B) ED668 (WT) and Δ*ckb1* (from the Deletion Mutant Library) cells were grown in EMM to OD_600_=0.5 and then transferred into EMM without Na_2_HPO_4_ (*n*=3). The mRNA levels of *ecl3*^+^ at 0, 3 and 6 h after phosphate starvation were measured by real-time PCR. Data show the mean±s.d. (C) ED668 (WT), ED668Δ*ecl1/2/3 h*^+^ (Δ*ecls*) and Δ*ckb1* (from the Deletion Mutant Library) cells were grown in YE to OD_600_=1.0 and then spotted on YE plates containing 5-fluorouracil (5-FU) with serial dilution (*n*=2). The Δ*ckb1* cells were spotted with two patterns of dilution because deletion of *ckb1*^+^ adversely affects cell growth even under unstressed conditions.

lncRNAs that regulate the PHO regulon are believed to be regulated by the HomolD box (Garg et al., 2018b) (Fig. 2A). Casein kinase 2 phosphorylates the HomolD-binding protein Rrn7 at Thr67, inhibiting the binding of Rrn7 to the HomolD box, causing the suppression of lncRNA transcriptional activity (Moreira-Ramos et al., 2015; Shuman, 2020). Casein kinase 2, which consists of the catalytic subunit α and the regulatory subunit β, is a widely conserved serine/threonine kinase essential for survival and proliferation and has several intracellular substrates (Homma, 2008). In *S. pombe*, *cka1*^+^ has been identified as encoding a catalytic subunit, and *ckb1*^+^ and *ckb2*^+^ have been identified as encoding regulatory subunits (Moreira-Ramos et al., 2015; Nakazawa et al., 2019; Romila et al., 2021). Although there are limited reports about the role of casein kinase 2 in fission yeast, Ckb1 has been suggested to be involved in regulating the Ca^2+^/calcineurin/Prz1 pathway (Ma et al., 2011). To explore whether *ecl3*^+^ expression is affected by lncRNAs, we investigated the level of *ecl3*^+^ during phosphate starvation in Δ*ckb1* cells. Phosphate starvation did not induce *ecl3*^+^ expression in Δ*ckb1* cells, indicating that the induction was dependent on Ckb1 (Fig. 2B). This supports the idea that the lncRNA-mediated PHO regulon, which is regulated through the HomolD box, also regulates the expression of *ecl3*^+^. In other words, Ckb1 deletion might suppress the phosphorylation of Rrn7 and promote the expression of lncRNAs, which in turn suppresses *ecl3*^+^ expression even under phosphate starvation.

It has been reported that Δ*ckb1* cells are sensitive to 5-fluorouracil (5-FU) (Mojardín et al., 2015). Unlike Δ*ckb1* cells, Δ*ecls* cells showed no significant 5-FU sensitivity (Fig. 2C). Moreover, consistent with previous reports, Δ*ckb1* cells showed poor growth even under non-stress conditions (Roussou and Draetta, 1994; Ma et al., 2011), but Δ*ecls* cells revealed no such growth retardation (Fig. 2C).

### Phosphate starvation might slightly extend CLS

Given that phosphate starvation induced the *ecl* family gene that regulates CLS, we investigated the effect of phosphate starvation on CLS. Both the prototroph JY1 and auxotroph JY333 strains exhibited much weaker, if any, CLS extension under phosphate starvation, which was significantly different from glucose restriction (calorie restriction), nitrogen starvation, leucine starvation and sulfur starvation (Fig. S4) (Ohtsuka et al., 2017, 2019; Imai et al., 2020). This might be attributable to the fact that phosphate starvation causes reduced induction of *ecl* family genes than other nutrient starvation conditions, such as leucine and sulfur starvation, which extend CLS in an *ecl* family gene-dependent manner; e.g. leucine starvation or sulfur starvation induces *ecl1*^+^ by approximately 100-fold (Ohtsuka et al., 2017, 2019, 2022d), whereas phosphate starvation induces *ecl3*^+^ by only approximately 20-fold, which is generally expressed at a lower level in comparison with *ecl1^+^*.

### Phosphate starvation induces sexual differentiation in a manner that is partially dependent on *ecl* family genes

**Fig. 3.**
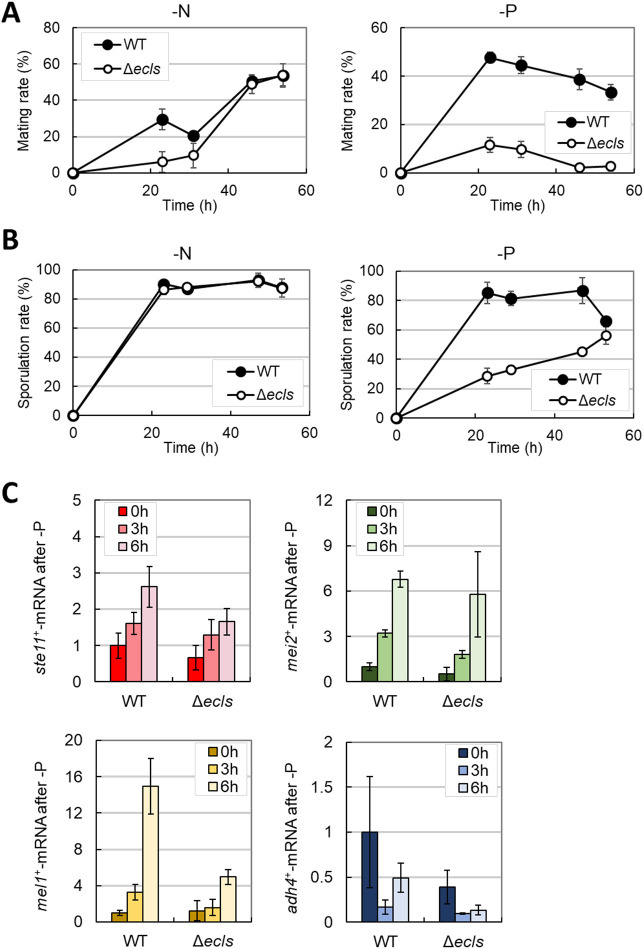
**Ecl1 family genes are required for proper sexual development under phosphate depletion.** (A) JY808 (WT) and JY808Δ*ecl1/2/3* (Δ*ecls*) cells were grown in EMM to OD_600_=0.5 and then transferred into nitrogen (−N) and phosphate (−P)-depleted EMM. Each mating rate was measured (*n*=3). (B) The diploid cells (JY333×HM3802) (WT) and JY333Δ*ecl1/2/3*×HM3802Δ*ecl1/2/3* (Δ*ecls*) were grown in EMM to OD_600_=0.5 and then transferred into nitrogen- and phosphate-depleted EMM. Each sporulation rate was measured (*n*=3). (C) JY333 (WT) and JY333Δ*ecl1/2/3* (Δ*ecls*) cells were grown in EMM to OD_600_=0.5 and then transferred into EMM without Na_2_HPO_4_ (*n*=3). The mRNA levels of *ste11*^+^, *mei2*^+^, *mel1*^+^ and *adh4*^+^ at 0, 3 and 6 h after phosphate starvation were measured using real-time PCR. Data show the mean±s.d.

The *ecl* family genes are also involved in sexual differentiation, that is, an overexpression of all *ecl* family genes including *ecl3*^+^ contributes to the induction of sexual differentiation (Ohtsuka et al., 2015, 2021d). Hence, we examined how *ecl* family genes affect sexual differentiation during phosphate starvation. The wild-type strain showed increased mating and sporulation rates under phosphate as well as nitrogen starvation (Fig. 3A,B; Fig. S5). Under nitrogen starvation, Δ*ecls* cells exhibited a slightly delayed mating rate and similar sporulation rate to that of wild-type cells. However, phosphate starvation led to dramatically decreased mating and delayed sporulation rates. This indicates that *ecl* family genes are essential for proper sexual differentiation under phosphate starvation.

It has been reported that the transcription factor Ste11 and RNA-binding protein Mei2 play crucial roles in sexual differentiation in *S. pombe* (Oowatari et al., 2011; Yamashita et al., 2017). As *ecl* family genes contribute to the expression of these factors (Ohtsuka et al., 2015), we examined their expression during phosphate starvation (Fig. 3C). Phosphate starvation altered the expression levels of *mel1*^+^, encoding a secreted α-galactosidase (Goddard et al., 2005), and *adh4*^+^, encoding an alcohol dehydrogenase (Sakurai et al., 2004), partially in an *ecl* family gene-dependent manner. However, the expression of *ste11*^+^ and *mei2*^+^ was induced irrespective of the presence or absence of *ecl* family genes. This result suggests that the essential function of *ecl* family genes in sexual differentiation during phosphate starvation is not the contribution to inducing the expression of genes such as *ste11*^+^ and *mei2*^+^.

### *ecl* family genes are required for proper G1 arrest during phosphate starvation

**Fig. 4.**
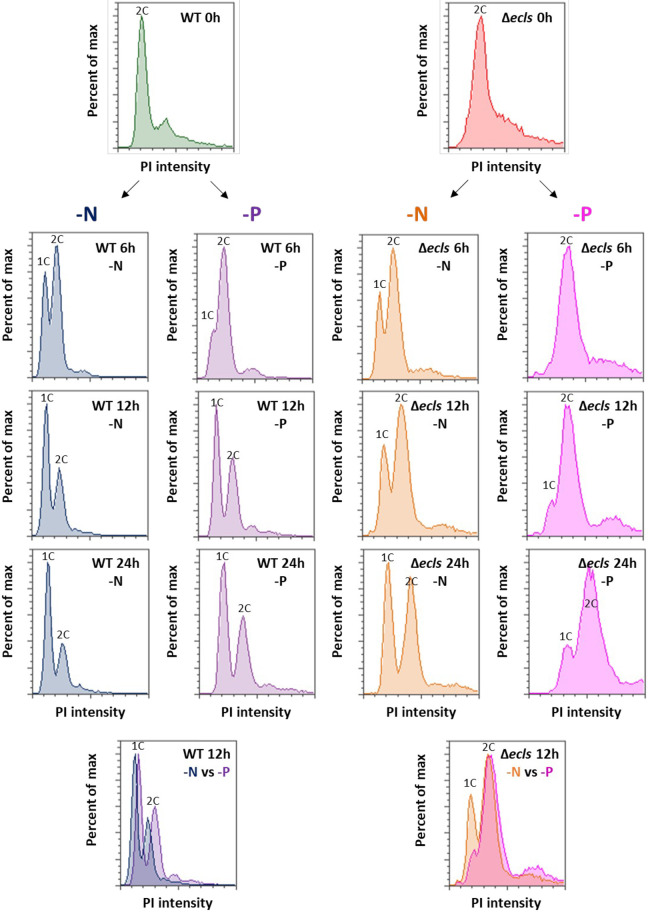
***ecl* family genes are required for proper G1 arrest under phosphate depletion.** JY333 (WT) and JY333Δ*ecl1/2/3* (Δ*ecls*) cells were grown in EMM to OD_600_=0.5 and then transferred into nitrogen- or phosphate-depleted EMM. DNA content was measured by flow cytometry. The merged results of 12 h after starvation are also shown in the bottom panels. All graphs show PI intensities from 0 to 100,000.

The phosphorylation of Ste11 by Cdc2 (CDK1) decreases its DNA-binding ability and then suppresses mating outside of the G1 phase; only G1 phase cells perform mating (Kjaerulff et al., 2007; Martín et al., 2020). Furthermore, the transcription factor Fkh2, which induces *ste11*^+^, is repressively phosphorylated by Cdc2, reinforcing the cell cycle regulation of mating (Shimada et al., 2008). We explored the possibility that *ecl* family genes affect mating through cell cycle control during phosphate starvation. To confirm this possibility, we observed the cell cycle during phosphate starvation using a flow cytometer. Phosphate starvation induced G1 arrest in wild-type cells, but the arrest was significantly delayed in Δ*ecls* cells, suggesting that *ecl* family genes are required for proper G1 arrest under phosphate starvation (Fig. 4). As reported previously (Ohtsuka et al., 2017), also under nitrogen starvation, the G1 arrest was slightly delayed in Δ*ecls* cells. These results support the notion that the decrease in mating rate in Δ*ecls* cells is due to the failure of proper G1 arrest under phosphate starvation.

### *ecl* family genes promote G1 arrest via the stabilization of Rum1

To elucidate how *ecl* family genes cause G1 arrest under phosphate starvation, we measured the protein amount of the CDK inhibitor Rum1, a regulator of Cdc2–cyclin B complexes that maintains low CDK activity in G1 and is required for G1 arrest under nitrogen starvation (Benito et al., 1998; Rubio et al., 2018; García-Blanco and Moreno, 2019). Moreover, under nutrient-rich conditions, Rum1 is phosphorylated and then degraded by SCF^Pop1/Pop2^ (Skp1–Cullin1–F-box), causing the increase of CDK activity and S-phase progression (MacKenzie and Lacefield, 2020; Stonyte et al., 2020). Under nitrogen starvation, Rum1 is stabilized, suppresses CDK activity and promotes G1 arrest.

**Fig. 5.**
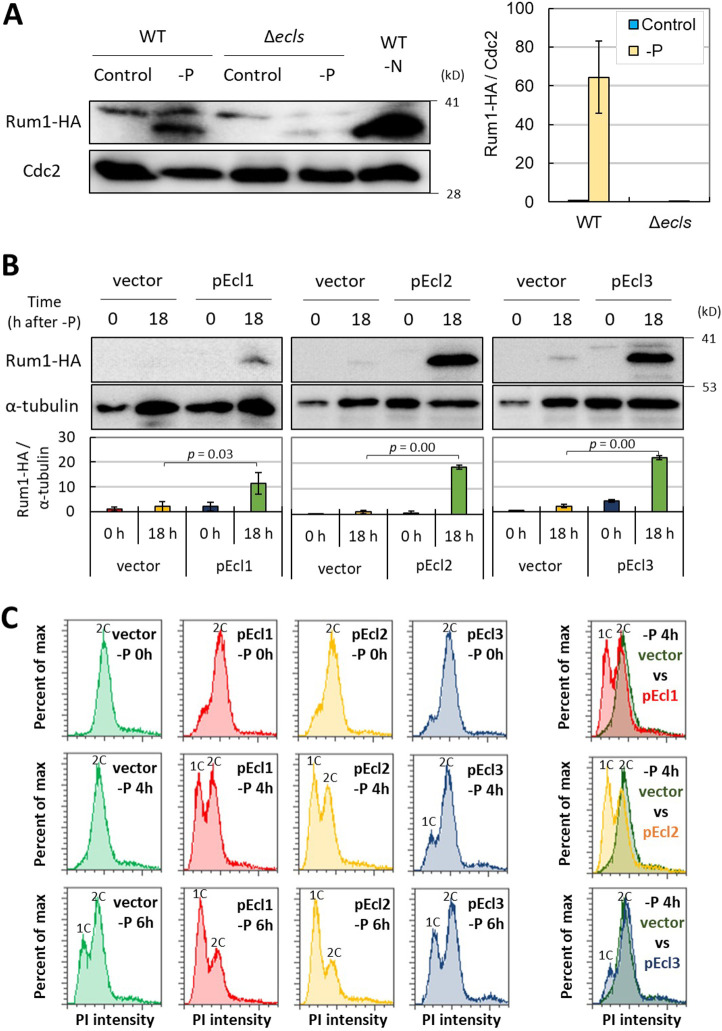
***ecl* family genes promote G1 arrest by stabilizing Rum1.** (A) WT (FY7288) and Δ*ecls* (FY7288Δ*ecl1/2/3*) cells were grown in EMM to OD_600_=0.5 (control) and then transferred into nitrogen-depleted (−N) or phosphate-depleted (−P) EMM for 24 h. The level of Rum1–HA was measured using western blot assay. Of the two bands detected by the anti-HA antibody, the upper band is expected to be phosphorylated (Benito et al., 1998; Matsuoka et al., 2002). The intensity of the Rum1–HA lower band was measured and normalized to the intensity of the Cdc2 band (Rum1–HA/Cdc2) (right panel) (*n*=3). (B) JY333 Rum1HA *h*^90^ cells carrying pREP1 (vector), pREP1-Ecl1 (pEcl1), pREP1-Ecl2 (pEcl2) and pREP1-Ecl3 (pEcl3) were grown in EMM to OD_600_=0.5 and then transferred into phosphate-depleted (−P) EMM for 18 h. The level of Rum1–HA was measured using western blot assay. The quantitative results are shown in the lower panels (*n*=3). (C) JY333 Rum1HA *h*^90^ cells carrying pREP1 (vector), pREP1-Ecl1 (pEcl1), pREP1-Ecl2 (pEcl2) and pREP1-Ecl3 (pEcl3) were grown in EMM to OD_600_=0.5 and then transferred into phosphate-depleted (−P) EMM. DNA content was measured via flow cytometry. The merged results of 4 h after starvation are shown in the right panels. All graphs show PI intensities from 0 to 50,000. Data show the mean±s.d.

Similar to nitrogen starvation, phosphate starvation also resulted in the accumulation of Rum1 in wild-type cells (Fig. 5A; Fig. S6), suggesting that Rum1 contributes to G1 arrest not only under nitrogen starvation but also under phosphate starvation. However, in the absence of *ecl* family genes, Rum1 accumulation was not detected even after 1 day of phosphate starvation (Fig. 5A), suggesting that *ecl* family genes contribute to Rum1 accumulation during phosphate starvation.

We next investigated whether overexpression of *ecl* family genes would promote Rum1 accumulation under phosphate starvation. The induction of each *ecl* family gene, namely *ecl1*^+^, *ecl2*^+^ and *ecl3*^+^, using plasmids with the *nmt1* promoter, resulted in Rum1 accumulation (Fig. 5B). This result supports the assertion that *ecl* family genes promote Rum1 accumulation.

Because the overexpression of *ecl* family genes resulted in Rum1 accumulation, we next investigated the cell cycle during phosphate starvation in cells with overexpression of *ecl* family genes. In control cells, G1-arrested cells were observed after 6 h of phosphate starvation, whereas in the *ecl* family gene-overexpressing cells, G1-arrested cells were observed from 4 h onward, indicating that *ecl* family genes promote G1 arrest (Fig. 5C). However, because G1-arrested cells were not detected before phosphate starvation in the *ecl* family gene-overexpressing cells, overexpression of *ecl* family genes alone was not sufficient to induce G1 arrest, suggesting that both *ecl* family genes and the starvation signal are required for G1 arrest. These data indicate that *ecl* family genes can promote G1 arrest by inducing the accumulation of Rum1 during phosphate starvation.

### Suppression of TORC1 does not restore Δ*ecls* phenotypes under phosphate starvation

Because Ecl family proteins reportedly suppress the activity of TORC1 under sulfur starvation (Ohtsuka et al., 2022a), the function of *ecl* family genes might be partially dependent on TORC1. Similar to the overexpression of *ecl* family genes, the suppression of TORC1 induces G1 arrest and sexual differentiation (Álvarez and Moreno, 2006). Hence, we investigated their involvement under phosphate starvation.

**Fig. 6.**
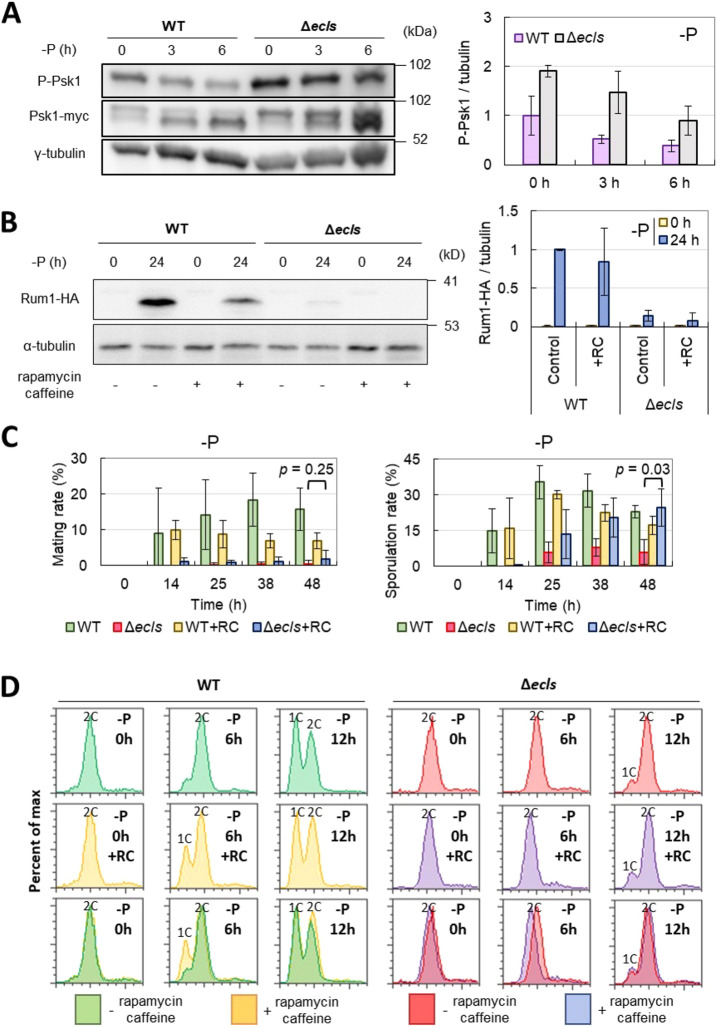
**Some phenotypes of Δ*ecls* cells were not restored by TORC1 suppression.** (A) JS183 (WT) and JS184 (Δ*ecls*) cells were grown in EMM to OD_600_=0.5 and then transferred into phosphate-depleted EMM. Phosphorylation levels of Psk1 at 0, 3 and 6 h after phosphate starvation (−P) were examined using western blotting (left). Quantitative results of phosphorylation levels (P-Psk1/γ-tubulin) are shown in the right panel (*n*=3). (B) WT (FY7288) and Δ*ecls* (FY7288Δ*ecl1/2/3*) were grown in EMM to OD_600_=0.5 (control) and then transferred into phosphate-depleted (−P) EMM (24 h) with and without 200 ng ml^−1^ rapamycin and 9 mM caffeine (RC). Rum1–HA levels were measured using western blotting (left). Quantitative results are shown in the right panel (*n*=3). (C) Left panel: JY808 (WT) and JY808Δ*ecl1/2/3* (Δ*ecls*) cells were grown in EMM to OD_600_=0.5 and then transferred into phosphate-depleted EMM with and without 200 ng ml^−1^ rapamycin and 9 mM caffeine (RC). Each mating rate was measured (*n*=3). Right panel: the diploid cells (JY333×HM3802) (WT) and JY333Δ*ecl1/2/3*×HM3802Δ*ecl1/2/3* (Δ*ecls*) were grown in EMM to OD_600_=0.5 and then transferred into phosphate-depleted EMM with and without 200 ng ml^−1^ rapamycin and 9 mM caffeine (RC). Each sporulation rate was measured (*n*=3). (D) JY333 (WT) and JY333Δ*ecl1/2/3* (Δ*ecls*) cells were grown in EMM to OD_600_=0.5 and then transferred into phosphate-depleted (−P) EMM with and without 200 ng ml^−1^ rapamycin and 9 mM caffeine. DNA content was measured by flow cytometry. All graphs show PI intensities from 0 to 50,000. Data show the mean±s.d.

First, to examine TORC1 activity during phosphate starvation, we measured the level of TORC1-added phosphorylation of Psk1, a TORC1 direct targets (Nakashima et al., 2012; Otsubo et al., 2017). We found that in the presence of *ecl* family genes, phosphate starvation decreased the phosphorylation of Psk1, suggesting the decreased activity of TORC1 (Fig. 6A). In contrast, the decrease in Psk1 phosphorylation was partially suppressed in the absence of *ecl* family genes. This indicates that *ecl* family genes can repress TORC1 under phosphate starvation. However, the decrease of phosphorylated Psk1 level appeared to be less under phosphate starvation than under sulfur starvation (Ohtsuka et al., 2022a). This might be because the expression of *ecl* family genes is less increased under phosphate starvation than under sulfur starvation (Ohtsuka et al., 2017). Moreover, it appears that the increase in total Psk1 protein level was observed under phosphate starvation (Fig. 6A), which might contribute to the increase in phosphorylated Psk1 level.

Because *ecl* family genes also repressed TORC1 under phosphate starvation, we next investigated the involvement of TORC1 in cellular responses that require *ecl* family genes, under phosphate starvation. As the CDK inhibitor Rum1 was stabilized in an *ecl* family gene-dependent manner under phosphate starvation (Fig. 5), we expected that TORC1 suppression could stabilize Rum1 even in Δ*ecls* cells, if TORC1 acts downstream of *ecl* family genes. Treatment with rapamycin and caffeine is known to inhibit TORC1 in fission yeast (Takahara and Maeda, 2012; Rallis et al., 2013). However, in our study, the reduction of Rum1 level in Δ*ecls* cells during phosphate starvation was not restored by treatment with rapamycin and caffeine (Fig. 6B). This implied that TORC1 suppression was not sufficient for Rum1 stabilization under phosphate starvation.

Next, we explored the involvement of TORC1 in sexual differentiation under phosphate starvation. Similar to the results shown in Fig. 3A, the *ecl* family genes were required for mating during phosphate starvation (Fig. 6C). Treatment with rapamycin and caffeine did not restore the mating defect of Δ*ecls* cells, suggesting that the mating defect of Δ*ecls* cells is not restored by TORC1 suppression and that *ecl* family genes are essential for mating under phosphate starvation. In contrast, Δ*ecls* cells showed a low sporulation rate as depicted in Fig. 3B, which was recovered by drug treatment (Fig. 6C). The reduction of the sporulation rate in Δ*ecls* cells might be partially caused by the defect of TORC1 suppression.

The *ecl* family genes might be required for G1 arrest under phosphate starvation, and, thus, although Δ*ecls* cells can sporulate from diploid cells, they cannot conjugate from haploid cells. Finally, we examined whether the defect of G1 arrest in Δ*ecls* cells could be restored by TORC1 suppression. We observed the cell cycles during phosphate starvation with rapamycin and caffeine treatment by flow cytometry (Fig. 6D). The defective G1 arrest in Δ*ecls* cells was not restored by treatment with these drugs. Conversely, these drugs hastened G1 arrest only in the presence of *ecl* family genes, suggesting that these genes are required for the promotion of G1 arrest by TORC1 suppression. Our data suggested that, during phosphate starvation, *ecl* family genes suppress TORC1 activity and are also required for appropriate cellular responses induced by TORC1 suppression, including G1 arrest.

## DISCUSSION

**Fig. 7.**
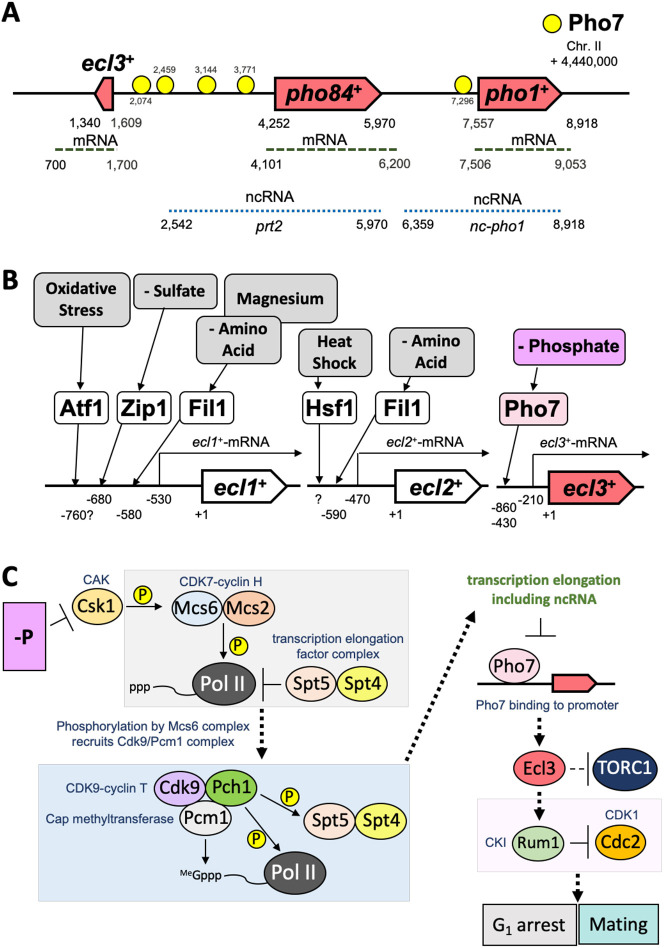
**Schematics for induction of *ecl* family genes.** (A) The chromosomal region from 4,440,000 to 4,450,000 of chromosome II containing *ecl3*^+^ in *S. pombe*. The binding sites of Pho7 are illustrated based on a previous study (Carter-O'Connell et al., 2012). ncRNA, noncoding RNA. (B) Signals and the transcription factors that induce *ecl* family genes. (C) Phosphate starvation induced sexual differentiation via the induction of Ecl family proteins. *S. pombe* has two partially redundant CDK-activating kinases (CAKs), namely, the CDK7–cyclin H (Mcs6–Mcs2 in *S. pombe*) complex and Csk1 (Hermand et al., 1998; Lee et al., 1999; Gerber et al., 2008). Under phosphate-rich conditions, the expression of PHO genes including *pho1*^+^ is suppressed through an active process involving Csk1, the positive transcription elongation factor b (P-TEFb) subunit Cdk9, and the phosphorylation of the carboxyl-terminal domain (CTD) of the Pol II catalytic subunit Rbp1 (Schwer et al., 2009; Sanchez et al., 2018). The CTD of Rpb1 undergoes dynamic phosphorylation changes during transcriptional elongation, leading to the recruitment of different factors, including capping enzymes, elongation factors and histone modifiers (Coudreuse et al., 2010; Yague-Sanz et al., 2020). Csk1 phosphorylates the transcription factor TFII-associated CDK Mcs6 (CDK7 ortholog) and contributes to the phosphorylation of the CTD of Rpb1 (Hermand et al., 1998; Lee et al., 1999; St. Amour et al., 2012). The catalytic activity of the CDK Mcs6 does not strictly depend on the cyclin component and it acts as a transcription factor TFII (TFIIH)-associated kinase (Lee et al., 1999; Viladevall et al., 2009). The Mcs6 complex phosphorylates the Pol II CTD and recruits the Cdk9 complex containing the cap methyltransferase Pcm1, resulting in further phosphorylation of Pol II CTD by the Cdk9 complex, pre-mRNA elongation and completion of the 5′-cap structure (Saha et al., 1999; Pei and Shuman, 2003; Viladevall et al., 2009; Coudreuse et al., 2010; St Amour et al., 2012; Paparidis et al., 2017). Furthermore, Cdk9 phosphorylates the CTD of the transcription elongation factor Spt5 and relieves Spt5-dependent elongation arrest (Pei and Shuman, 2003; Viladevall et al., 2009; Schwer et al., 2009; Parua et al., 2018). The complex consisting of Spt5 and Spt4 regulates the early transcriptional elongation of Pol II and acts on pre-mRNA processing through physical interactions with mRNA-capping enzymes (Schwer et al., 2009). CKI: CDK inhibitor.

This study demonstrated that phosphate starvation induces *ecl3*^+^ expression and *ecl* family genes contribute to appropriate G1 arrest and sexual differentiation under phosphate starvation. Moreover, the induction of *ecl3*^+^ was significantly dependent on the transcription factor Pho7 (Fig. 1B). *ecl3*^+^ is located at the bases 4,441,609–4,441,340 of chromosome II, and *pho84*^+^ and *pho1*^+^, which act in the PHO pathway, are located upstream of its 5′ side (Fig. 7A). All these genes are regulated by Pho7, and there are multiple Pho7-binding sites in the chromosome region from *ecl3*^+^ to *pho1*^+^ (Carter-O'Connell et al., 2012). Because there are also Pho7-binding sites upstream of *ecl3*^+^, Pho7 is considered to directly induce the transcription of *ecl3*^+^ in the same manner as that by *pho1*^+^ and *pho84*^+^.

In *S. pombe*, *ecl* family genes are induced in response to various starvation conditions and environmental stresses (Ohtsuka and Aiba, 2017) (Fig. 7B). Because Ecl1, Ecl2 and Ecl3 have similar amino acid sequences, all of them can complement the Δ*ecls* phenotype, and each elicits similar intracellular responses, so their molecular functions are also probably similar (Ohtsuka et al., 2015; Ohtsuka and Aiba, 2017). Using these three *ecl* family genes, *S. pombe* might induce appropriate intracellular responses to various environmental stresses and nutritional starvations, leading to the maintenance of cell survival, including CLS extension and induction of sexual differentiation. Although environmental stresses and nutritional starvation are extremely diverse, and because some of these stimuli are transmitted and converged to *ecl* family genes, it is expected that the *S. pombe* cell can exhibit similar cellular responses to various environmental stimuli.

Phosphate starvation results in a G1 arrest that is mediated by *ecl* family genes (Figs 4 and 5C). Similar to phosphate starvation, amino acid starvation causes cells to undergo G1 arrest, and this arrest also depends on *ecl* family genes (Ohtsuka et al., 2019). However, sulfur starvation, as well as phosphate or amino acid starvation, also induces *ecl* family genes, but cells undergo arrest in the G2 phase (Ohtsuka et al., 2017). The CDK inhibitor Rum1 plays a vital role in the G1 arrest of cells (Stonyte et al., 2020). In this study, we found that Rum1 stabilization is essential for proper G1 arrest during phosphate starvation and that *ecl* family genes contribute to this (Fig. 5). In contrast, during sulfur starvation, Rum1 is degraded even in the presence of *ecl* family genes (Ohtsuka et al., 2022a). This indicates that *ecl* family genes contribute to the stabilization of Rum1, but the induction of *ecl* family genes alone is not sufficient to stabilize Rum1. This does not contradict the fact that the overexpression of *ecl* family genes alone did not cause either Rum1 stabilization or G1 arrest (0 h in Fig. 5B,C), and both the presence of *ecl* family genes and phosphate starvation were required for their responses. It is not yet known how Ecl family proteins stabilize Rum1 under phosphate starvation.

Similar to other PHO regulon genes, *ecl3*^+^ expression was upregulated in Δ*csk1* cells even under phosphate-replete conditions and was induced to a lower degree by phosphate starvation (Fig. 1C). Moreover, *ecl3*^+^ is located adjacent to a PHO gene, *pho84*^+^, suggesting that the mechanism of *ecl3*^+^ induction is extremely similar to that of other PHO regulon genes (Sanchez et al., 2019; Garg et al., 2020; Schwer et al., 2021). Studies have also reported that regulation of the PHO regulon in *S. pombe* is mediated by upstream lncRNAs regulated by C-terminal domain (CTD) phosphorylation of Pol II, which is partially regulated by Csk1 via Cdk9, and by silencing through the methylation of histone H3 Lys9 at PHO gene regions, causing the inhibition of Pho7-binding to the promoter site of PHO genes (Shah et al., 2014; Yague-Sanz et al., 2020). The result that *ecl3*^+^ expression was regulated not only by Pho7 but also by Csk1 supports the idea that *ecl3*^+^ is regulated by the PHO regulon. In addition, consistent with this idea, the deletion of Ckb1, the regulatory subunit of casein kinase 2, which mediates the HomolD box-regulated transcription, including the lncRNA of PHO regulon, did not induce *ecl3*^+^ under phosphate starvation.

Various factors, such as Csk1, Pho7, Ecl family proteins, including Ecl3, and Rum1, are considered to be involved in sequential cellular processes, ranging from perception of phosphate starvation to conjugation (Fig. 7C). Csk1 might recruit Cdk9 to the Pol II complex through the phosphorylation of Mcs6; Cdk9 phosphorylates the Pol II CTD and the Spt5–Spt4 complex, resulting in the alleviation of Pol II elongation arrest at the promoter proximal position (Hermand et al., 1998; Pei and Shuman, 2003; Viladevall et al., 2009; Schwer et al., 2009). Thus, it has been suggested that transcriptional elongation is stimulated through reactions involving the phosphorylation of Pol II CTD by Mcs6 and Cdk9 (Coudreuse et al., 2010). In contrast, a non-Mcs6-mediated pathway has also been proposed for the action of Csk1 on Cdk9 (Gerber et al., 2008). It is believed that the Csk1-mediated regulation of transcriptional elongation also targets the lncRNA located upstream of PHO genes, and the inhibition of Csk1 suppresses lncRNA elongation (Carter-O'Connell et al., 2012; Estill et al., 2015; Sanchez et al., 2018). Insufficient elongation of lncRNAs might allow the binding of Pho7 to the upstream sequences of PHO genes, including *ecl3*^+^. Our data suggested that the induction of *ecl3*^+^ by Pho7 causes stabilization of Rum1 and suppression of Cdc2 activity, promoting G1 arrest. Simultaneously, the suppression of Cdc2, which phosphorylates and inhibits factors related to sexual differentiation, such as Ste11 and Fkh2 (Kjaerulff et al., 2007; Shimada et al., 2008), is also considered to cause sexual differentiation in fission yeast.

## MATERIALS AND METHODS

### Strains and growth media

*S. pombe* strains are listed in Table S1. The *S. pombe* Deletion Mutant Library from Bioneer (http://us.bioneer.com/products/spombe/spombeoverview.aspx) was also used. The correct genotypes of the deletion mutants have been tested by using appropriate primers with PCR. Cells were grown in EMM supplemented with essential nutrients (Takuma et al., 2013). For nitrogen- and phosphate-depleted media, NH_4_Cl and Na_2_HPO_4_ were omitted from EMM, respectively. The amounts of supplemental nutrients were as follows: 40 µg ml^−1^ adenine, 60 µg ml^−1^ leucine and 20 µg ml^−1^ uracil. Cells were grown at 30°C unless otherwise stated.

### Plasmids

The following plasmids were used: pLB-Dblet (Ohtsuka et al., 2009), pEcl3 (Ohtsuka et al., 2009), pREP1 (Ohtsuka et al., 2008), pREP1-Ecl1 (Ohtsuka et al., 2008), pREP1-Ecl2 (Ohtsuka et al., 2009) and pREP1-Ecl3 (Ohtsuka et al., 2009).

### Construction of the FY7288Δ*ecl1/2/3*, ED668*Δecl1/2/3 h^+^*, JY333Δ*csk1*, JY333Δ*pho7* and JY333 Rum1HA *h^90^* strains

The FY7288Δ*ecl1/2/3* and JY333 Rum1HA *h*^90^ strains were generated by mating cells of the FY7288 strain from the National Bio-Resource Project, Japan, with cells of the JY333Δ*ecl1/2/3* strain (Ohtsuka et al., 2015). The ED668Δ*ecl1/2/3 h*^+^ strain was generated by mating cells of the ED668 strain with cells of the JY333Δ*ecl1/2/3* strain (Ohtsuka et al., 2015). The JY333Δ*csk1* and JY333Δ*pho7* strains were generated by mating cells of the JY333 strain with cells of Δ*csk1* and Δ*pho7* strains from the *S. pombe* Deletion Mutant Library from Bioneer, respectively. The correct genotypes of these strains have been tested using appropriate primers with PCR.

### Construction of the JS183 and JS184 strains

To construct JS183, we fused a 13MYC-tag to the C-terminus of the Psk1 protein on the chromosomes of JY1 as described previously (Sato et al., 2005). For *ecl1*^+^, *ecl2*^+^ and *ecl3*^+^ disruptions, the ORF regions of Ecl proteins were replaced with kan^r^, nat^r^ and bsd^r^ cassettes, respectively, using previously described methods (Sato et al., 2005; Fujita et al., 2015). The primers used for this purpose are listed in Table S1. The correct genotypes of these strains have been tested using appropriate primers with PCR.

### Measurement of mating and sporulation rates

All data were calculated by counting at least 300 cells. The mating and sporulation rates were calculated as described previously (Ohtsuka et al., 2015).

### Real-time PCR analysis

Real-time PCR analysis was performed as described previously (Hibi et al., 2018) using the housekeeping gene *cdc2*^+^ as a control. The primers are listed in Table S1.

### Western blot analysis

Western blotting was performed as described previously (Hibi et al., 2018). Rum1–HA was detected with the anti-HA (12CA5) antibody (1:10,000; Roche, 11666606001), Cdc2 with the anti-CDK1 antibody (Y100.4) (1:10,000; Abcam, ab5467), Psk1–myc with the MYC/c-Myc antibody (1:2000; Santa Cruz Biotechnology, sc-40), phospho-Psk1-myc with the anti-phospho-Psk1/RPS6KB1/p70 S6 kinase (Thr389) antibody (1:1000; Cell Signaling Technology, 9206), γ-tubulin with the monoclonal anti-Gtb1/γ-tubulin antibody (1:2000; Sigma-Aldrich, T5326), and α-tubulin with the monoclonal anti-α-tubulin antibody (1:10,000; Sigma-Aldrich, T6074).

### Flow cytometry analysis

The cells were fixed with 70% ethanol and treated with an RNase-solution (Sigma-Aldrich) to measure the DNA content for 1 h at 37°C. Nucleic acids were stained with propidium iodide (PI). Flow cytometry analysis was performed using the Attune Acoustic Focusing Cytometer (Life Technologies). More than 4000 cells were examined for each sample; the vertical axis of the graph indicates the maximum percentage, and the maximum values of the peak are 100%. The PI intensity was detected at a voltage of 2.2 V on the BL2 channel.

### Statistical analysis

Quantitative data shown in figures represent the average of at least three independent experiments ±s.d. Statistical analyses were performed by two-tailed unpaired Student's *t*-test.

## Supplementary Material

10.1242/joces.260759_sup1 Supplementary information
