# Supplementary material for: The ecl family gene ecl3+ is induced by phosphate starvation and contributes to sexual differentiation in fission yeast
Source: J Cell Sci. 2023 Mar 10;136(6):jcs260759. doi: 10.1242/jcs.260759 (PMC10038150; doi:10.1242/jcs.260759)
Supplement: Supplementary information [file joces-136-260759-s1.pdf]

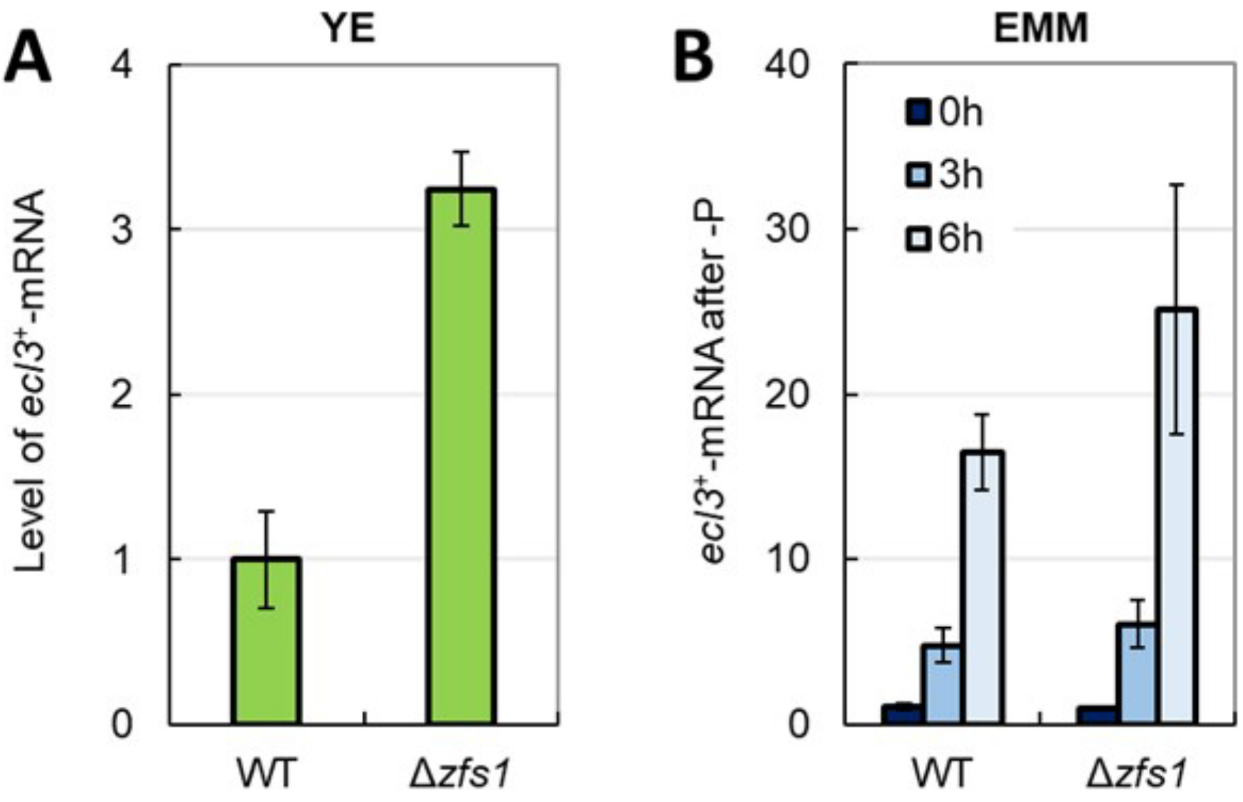

Fig. S1. ***ecl3*$^{+}$ was induced by phosphate starvation in the absence of Zfs1.** (**A**) JY333 (WT) and JZ974 (Δ*zfs1*)(Kanoh et al. 1995) cells were grown in YE medium to OD_{600} = 0.5, and the mRNA levels of *ecl3*$^{+}$ were measured by real-time PCR assay (n = 3). (**B**) JY333 (WT) and JZ974 (Δ*zfs1*) cells were grown in EMM to OD_{600} = 0.5 and then transferred into EMM without Na_2HPO_4 (n = 3).

Journal of Cell Science • Supplementary information

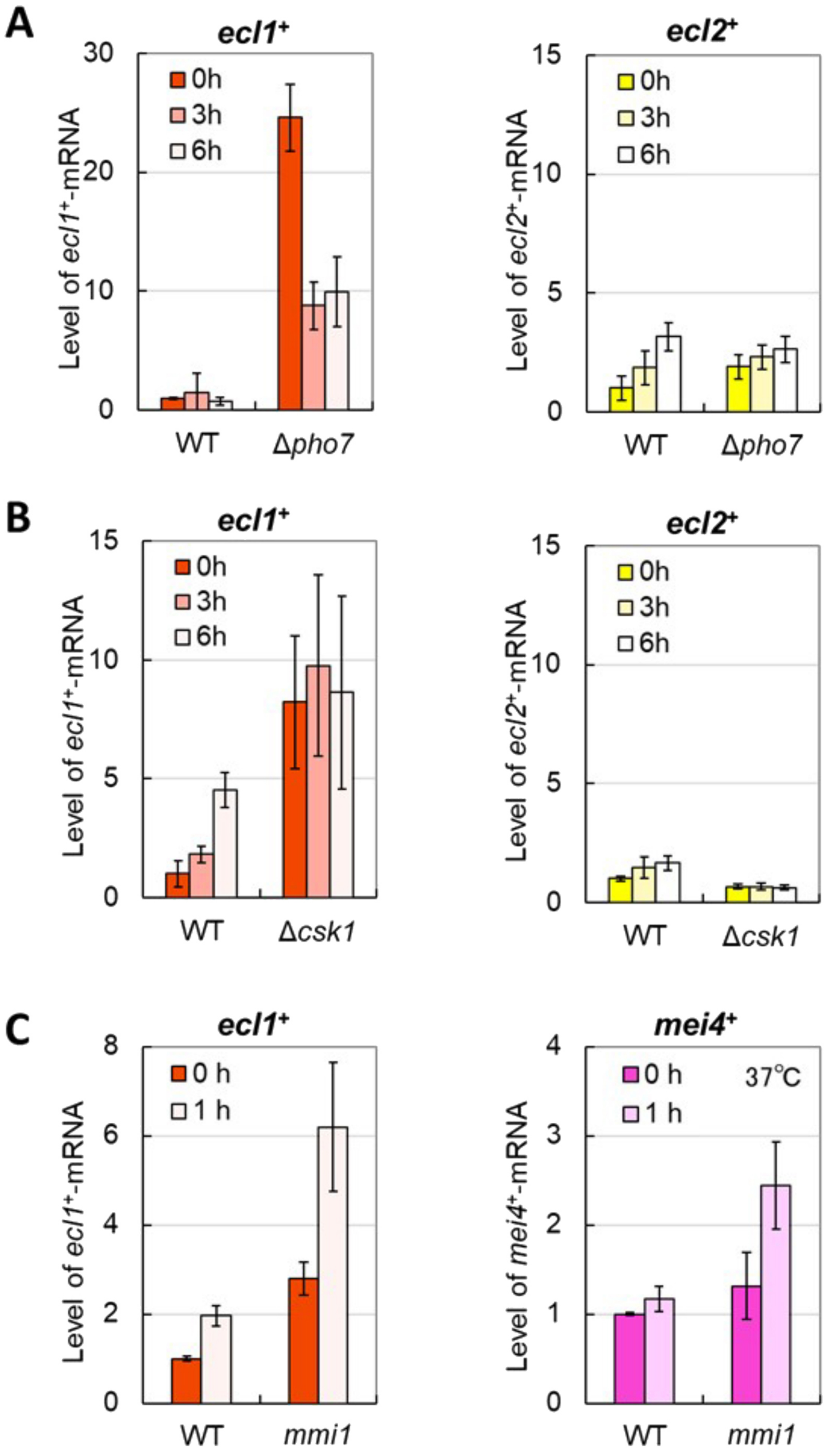

Fig. S2. The mRNA levels of ***ecl1***$^{+}$ **and** ***ecl2***$^{+}$ **were measured by real-time PCR assay.** (A) JY333 (WT) and JY333Δ*pho7* (Δ*pho7*) cells were grown in EMM to OD_{600} = 0.5 and then transferred into EMM without Na2HPO4 (n = 3). (B) JY333 (WT) and JY333*Δcsk1* (*Δcsk1*) cells were grown in EMM to OD_{600} = 0.5 and then transferred into EMM without Na2HPO4 (n = 3). (C) JY450 (WT) and JV579 (mmi1)(Yamashita et al. 2012) cells were grown in YE to OD600 = 0.5 at 25°C and then transferred to 36°C for 1 h (n = 3). For cultures of JY333, JY333*Δpho7*, JY333*Δcsk1*, JY450, and JV579 cells, 40 mg L^{-1} adenine and 60 mg L^{-1} leucine were added.

Journal of Cell Science • Supplementary information

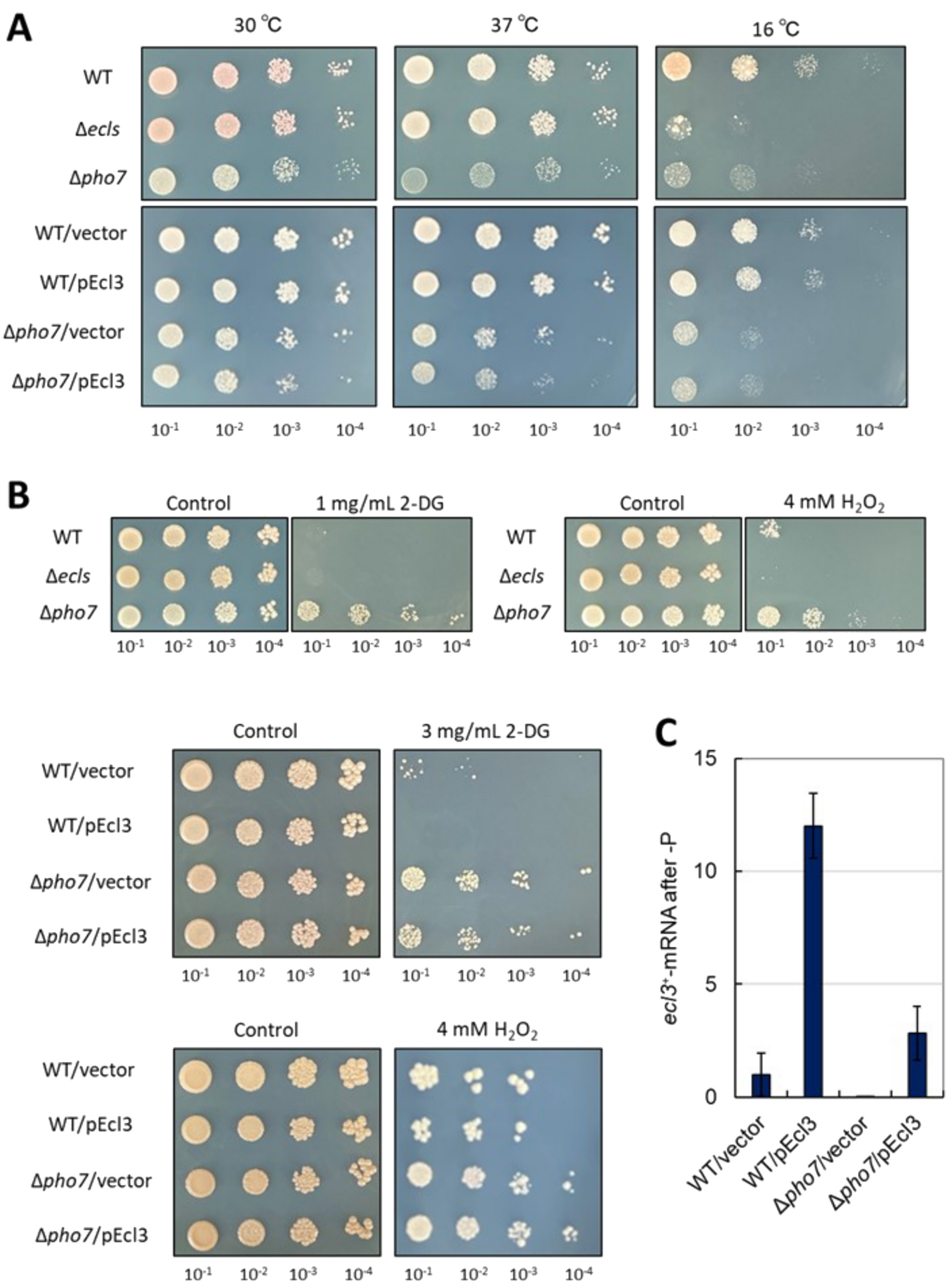

Fig. S3. Effects of *ecl* family genes and *pho7*+ on various stress sensitivities. (**A**) JY333 (WT), JY333Δ*ecl1/2/3* (Δ*ecls*), and JY333Δ*pho7* (Δ*pho7*) cells were grown in YE to OD_{600} = 1.0 and then spotted on YE plates with serial dilution (upper panel). JY333/pLB-Dblet (WT/vector), JY333/pEcl3 (WT/pEcl3), JY333Δ*pho7*/pLB-Dblet (Δ*pho7*/vector), and JY333Δ*pho7*/pEcl3 (Δ*pho7*/pEcl3) cells were grown in EMM to OD_{600} = 1.0 and then spotted on EMM plate with serial dilution (lower panel). The plates were incubated at 16°C, 30°C, and 37°C. (**B**) JY333 (WT), JY333Δ*ecl1/2/3* (Δ*ecls*), and JY333Δ*pho7* (Δ*pho7*) cells were grown in YE to OD_{600} = 1.0 and then spotted on YE plates containing 2-DG or H_2O_2 with serial dilution (upper panel). JY333/ pLB-Dblet (WT/vector), JY333/pEcl3 (WT/pEcl3), JY333Δ*pho7*/pLB-Dblet (Δ*pho7*/vector), and JY333Δ*pho7*/pEcl3 (Δ*pho7*/pEcl3) cells were grown in EMM to OD_{600} = 1.0 and then spotted on EMM plates containing 2-DG or H_2O_2 with serial dilution (lower panel). (**C**) JY333/pLB-Dblet (WT/vector), JY333/pEcl3 (WT/pEcl3), JY333Δ*pho7*/pLB-Dblet (Δ*pho7*/vector), and JY333Δ*pho7*/pEcl3 (Δ*pho7*/pEcl3) cells were grown in EMM to OD_{600} = 0.5. The mRNA level of *ecl3*+ was measured (*n* = 3).

Journal of Cell Science • Supplementary information

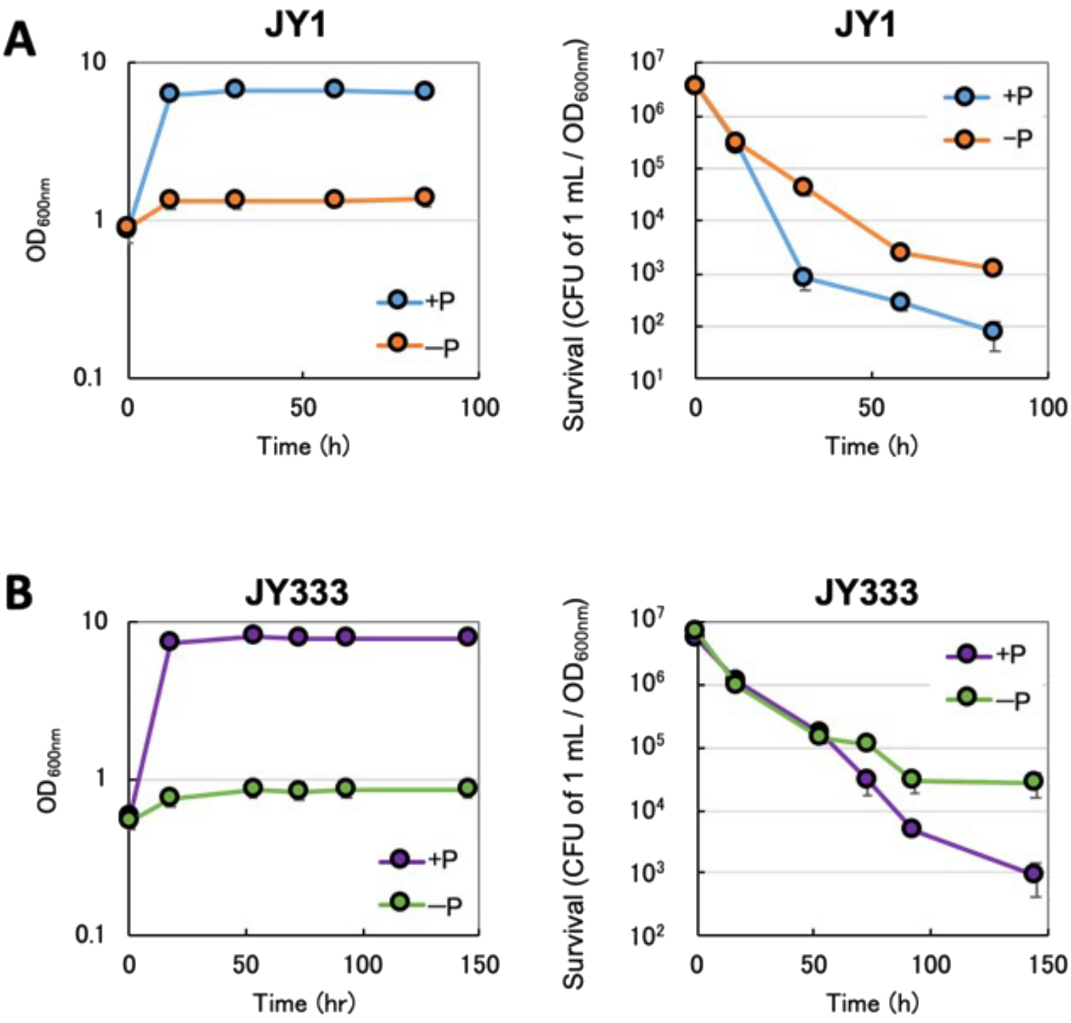

Fig. S4. The growth (left panels) and CLS (right panels) were measured during phosphate starvation. (**A**) JY1 cells were cultured in synthetic SD medium (Ohtsuka et al. 2017) with or without KH_2PO_4 (*n* = 3). (**B**) JY333 cells were cultured in synthetic SD medium with or without KH_2PO_4 (*n* = 3). Colony-forming units were used to measure survival (Ohtsuka et al. 2017).

Journal of Cell Science • Supplementary information

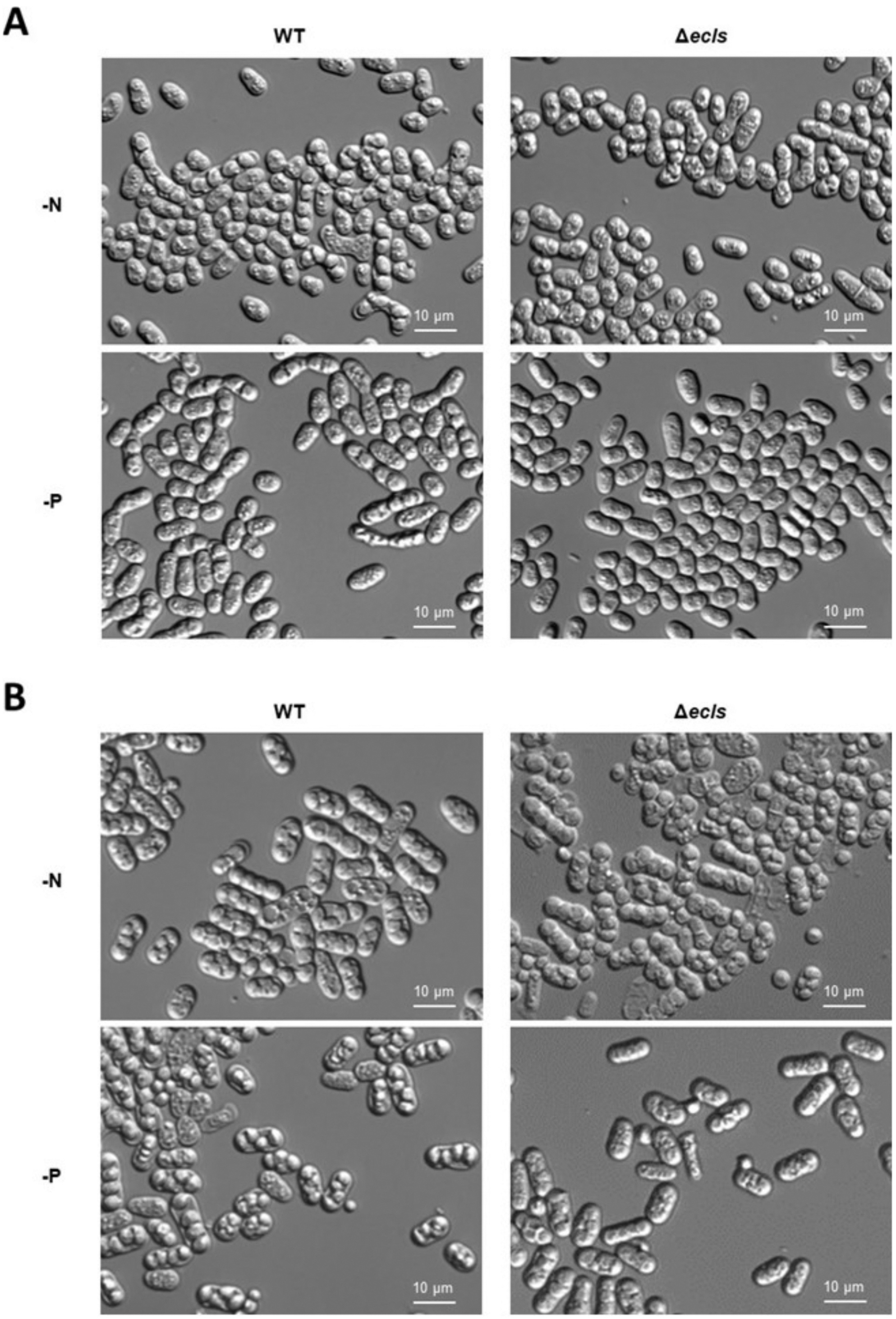

Fig. S5. Sexual development under phosphate depletion. (**A**) JY808 (WT) and JY808Δ*ecl1/2/3* (Δ*ecls*) cells were grown in EMM to OD_{600} = 0.5 and then transferred into nitrogen- and phosphate-depleted EMM for 25 h. (**B**) The diploid cells (JY333 × HM3802) (WT) and (JY333Δ*ecl1/2/3* and HM3802Δ*ecl1/2/3*) (Δ*ecls*) were grown in EMM to OD_{600} = 0.5 and then transferred into nitrogen- and phosphate-depleted EMM for 25 h.

Journal of Cell Science • Supplementary information

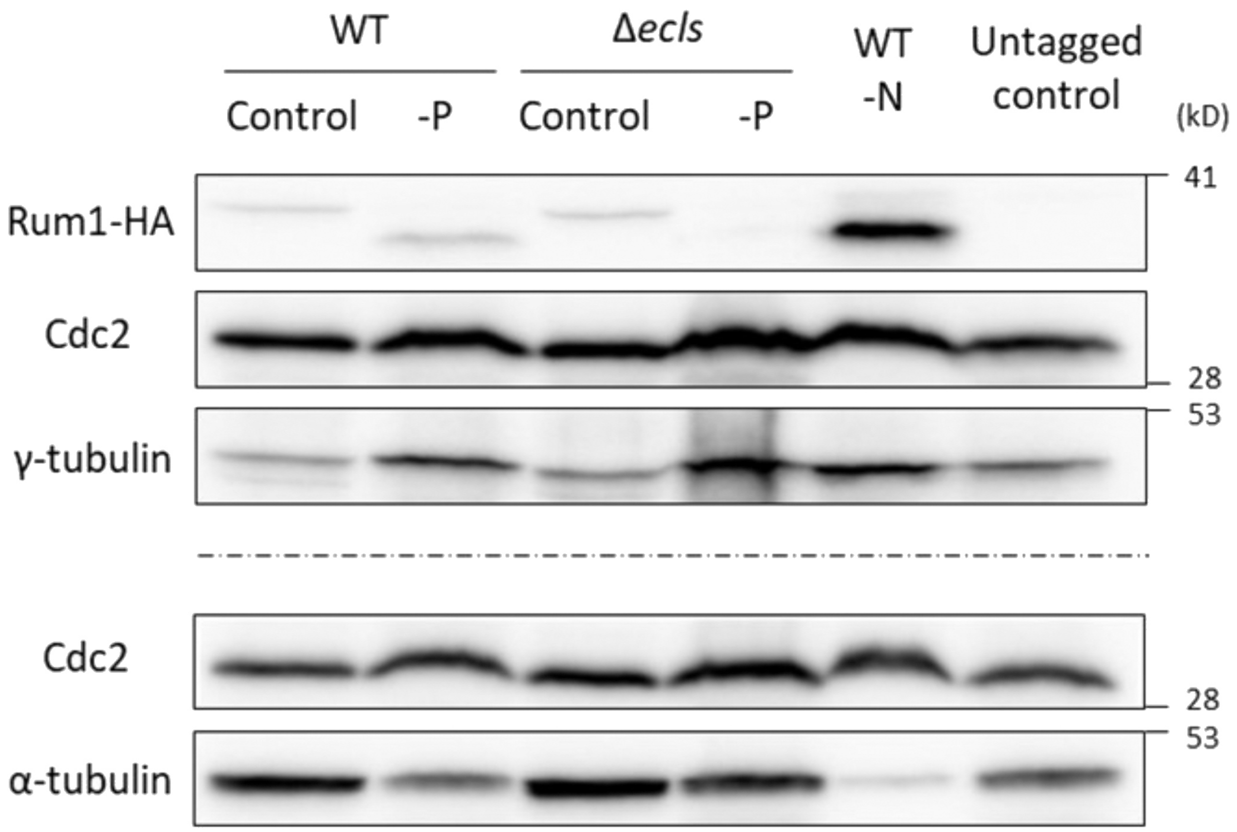

Fig. S6. Rum1 levels under phosphate depletion. WT (FY7288) and Δ*ecls* (FY7288Δ*ecl1/2/3*) were grown in EMM to OD_{600} = 0.5 (Control) and then transferred into nitrogen-depleted (−N) or phosphate-depleted (−P) EMM (18 h). The level of Rum1-HA was measured using western blot assay. Both upper and lower bands of Rum1-HA were not observed in the untagged control, suggesting that these bands were derived from Rum1-HA.

Table S1. Yeast Strains and PCR Primers

Click here to download Table S1

References

Kanoh, J., Sugimoto, A. and Yamamoto, M. (1995). *Schizosaccharomyces pombe zfs1+* encoding a zinc-finger protein functions in the mating pheromone recognition pathway. *Mol. Biol. Cell* **6**, 1185-1195. doi:10.1091/mbc.6.9.1185

Yamashita, A., Sakuno, T., Watanabe, Y. and Yamamoto, M. (2017). Analysis of Schizosaccharomyces pombe meiosis. *Cold Spring Harb. Protoc.* 2017, pdb.top079855. doi:10.1101/pdb.top079855

Journal of Cell Science • Supplementary information
